# Atherosclerosis profile and incidence of cardiovascular events: a population-based survey

**DOI:** 10.1186/1471-2261-9-46

**Published:** 2009-09-15

**Authors:** Jennifer G Robinson, Kathleen M Fox, Michael F Bullano, Susan Grandy

**Affiliations:** 1 Department of Medicine, University of Iowa, Iowa City, IA, USA; 2 Strategic Healthcare Solutions, LLC, Monkton, MD, USA; 3 Health Economics and Outcomes Research, AstraZeneca Pharmaceuticals LP, Wilmington, DE, USA

## Abstract

**Background:**

Atherosclerosis is a chronic progressive disease often presenting as clinical cardiovascular disease (CVD) events. This study evaluated the characteristics of individuals with a diagnosis of atherosclerosis and estimated the incidence of CVD events to assist in the early identification of high-risk individuals.

**Methods:**

Respondents to the US SHIELD baseline survey were followed for 2 years to observe incident self-reported CVD. Respondents had subclinical atherosclerosis if they reported a diagnosis of narrow or blocked arteries/carotid artery disease without a past clinical CVD event (heart attack, stroke or revascularization). Characteristics of those with atherosclerosis and incident CVD were compared with those who did not report atherosclerosis at baseline but had CVD in the following 2 years using chi-square tests. Logistic regression model identified characteristics associated with atherosclerosis and incident events.

**Results:**

Of 17,640 respondents, 488 (2.8%) reported having subclinical atherosclerosis at baseline. Subclinical atherosclerosis was associated with age, male gender, dyslipidemia, circulation problems, hypertension, past smoker, and a cholesterol test in past year (OR = 2.2) [all p < 0.05]. Incident CVD was twice as high in respondents with subclinical atherosclerosis (25.8%) as in those without atherosclerosis or clinical CVD (12.2%). In individuals with subclinical atherosclerosis, men (RR = 1.77, p = 0.050) and individuals with circulation problems (RR = 2.36, p = 0.003) were at greatest risk of experiencing CVD events in the next 2 years.

**Conclusion:**

Self-report of subclinical atherosclerosis identified an extremely high-risk group with a >25% risk of a CVD event in the next 2 years. These characteristics may be useful for identifying individuals for more aggressive diagnostic and therapeutic efforts.

## Background

Clinical manifestations of atherosclerosis, including coronary artery disease, cerebrovascular disease, and peripheral arterial disease, will occur in 2 of 3 men and 1 in 2 women after age 40. [1] Almost 60% of deaths are due to a cardiovascular disease (CVD) cause. Atherosclerosis is a chronic, progressive disease with a long asymptomatic phase. [2] Subclinical atherosclerosis is a latent precursor of clinical CVD, including myocardial infarction (MI) and stroke. [3]

The Healthy People 2010 initiative and the Public Health Action Plan to Prevent Heart Disease and Stroke call for improving cardiovascular health through prevention, detection, and treatment of risk factors, as well as prevention of recurrent CVD events. [4-6] The American Heart Association (AHA) guidelines for primary and secondary prevention of atherosclerotic CVD provide evidence and recommendations to assist clinicians in managing and treating atherosclerosis. [7-9] However, evidence is limited on identifying individuals with subclinical atherosclerosis before a CVD event occurs, especially since specialized diagnostic imaging is not part of routine clinical practice. The purpose of the present study was to evaluate the self-reported characteristics and estimate the incidence of CVD events among individuals with subclinical atherosclerosis to assist in the early identification of high-risk individuals.

## Methods

A cohort of individuals with a diagnosis of subclinical atherosclerosis with no prior history of CVD events (i.e., MI, stroke, or revascularization) was identified from the **S**tudy to **H**elp **I**mprove **E**arly evaluation and management of risk factors **L**eading to **D**iabetes (SHIELD) and compared with SHIELD individuals without reported atherosclerosis and no prior history of CVD events. SHIELD included an initial screening phase to identify cases of interest in the general population and a detailed baseline survey to follow up identified cases for health status, health knowledge, attitudes, behaviors, and treatment. Annual follow-up surveys were administered to obtain changes in health status, behaviors and treatment. A detailed description of the SHIELD methodology has been published previously. [10,11]

In brief, the screening survey was mailed to a stratified random sample of 200,000 US households, representative of the US population for geographic residence, household size and income, and age of head of household. [12] The head of household provided responses for up to 4 adult (aged ≥18 years) household members, resulting in a response rate of 63.7% (127,420 households for 211,097 adults). The baseline survey was sent in 2004 to 22,001 individuals, a stratified random sample of screening respondents, who self-reported diabetes mellitus or cardiometabolic risk factors. Each respondent group was balanced to be representative of that population (e.g. type 1 diabetes, type 2 diabetes) for age, gender, geographic region, household size and income as the US population. A response rate of 71.8% was obtained (n = 15,794).

In August 2005, the first annual follow-up survey was mailed to all individuals selected for the baseline survey who were still enrolled in the household panel (n = 19,613). The second follow-up survey was mailed in July 2006 to individuals who had returned either the baseline and/or first annual questionnaires (n = 18,445). The 2005 survey had a response rate of 72%, and a 75% response rate was obtained for the 2006 survey (n = 13,877). This study utilized the baseline, 2005, and 2006 survey responses.

### Study Population

Respondents to the baseline survey were considered to have subclinical atherosclerosis if they reported a diagnosis of narrow or blocked arteries or carotid artery disease without a past clinical CVD event (heart disease, heart attack, stroke, angioplasty, or heart bypass surgery) based on the survey question of "have you ever been told by a doctor, nurse or other health professional that you have narrow or blocked arteries/carotid artery disease". Respondents were also asked if they "had ever been told that you have heart disease/heart attack, stroke/TIA or had angioplasty, stents or surgery to clear your arteries or heart bypass surgery". The comparison cohort for the cross-sectional analysis was composed of individuals who did not report a diagnosis of atherosclerosis (subclinical) and/or clinical CVD (i.e. no prior history of CVD event) in the baseline survey.

### CVD Events

CVD events included heart attack, stroke, angioplasty, or heart bypass surgery. An incident CVD event was defined as a new CVD event reported in either the 2005 or 2006 survey without a history of a CVD event from the baseline survey. Respondents who completed the baseline, 2005, and 2006 surveys and responded positively to the survey questions for atherosclerosis and CVD events (n = 7,483) were included in the analysis of incident CVD events.

Respondents were asked to provide their weight and height for calculation of body mass index. They also were provided a measuring tape and instructed that while standing, hold the tape measure loosely around their waist at the level of their navel ("belly button") to determine waist circumference. Respondents were asked whether they "had ever been told by a doctor, nurse or health professional that you had circulation problems of any kind (categorized as circulation problems) or cholesterol problems" (categorized as dyslipidemia).

### Statistical Analysis

Two separate analyses were conducted: 1) cross-sectional analysis of baseline respondents for prevalence and characteristics predicting atherosclerosis, and 2) longitudinal analysis of incident CV events. Prevalence of subclinical atherosclerosis was calculated as the number of individuals reporting a diagnosis of atherosclerosis at the baseline survey divided by the total number of respondents to the baseline survey. Incidence of CVD events was quantified as the number of new CVD events that were reported during the 2 years of follow-up since the baseline survey. Demographic features, comorbid conditions, obesity, and smoking history of individuals with subclinical atherosclerosis were compared with those of the cohort who did not report a diagnosis of atherosclerosis or previous CVD event, using two-sided chi-square tests. Similar comparisons were made between respondents with atherosclerosis and incident CVD events and the respondents with no atherosclerosis but who had an incident CVD event.

Logistic regression analyses identified characteristics associated with atherosclerosis without prior history of CVD event and atherosclerosis with incident CVD events. To identify characteristics of subclinical atherosclerosis, the regression model included individuals with and without subclinical atherosclerosis from the baseline survey as the dependent variable and age, gender, race, household income, education, geographic region, dyslipidemia, circulation problems, diabetes mellitus, hypertension, abdominal obesity, smoking, and cholesterol test in past year as independent variables. To identify characteristics of those with subclinical atherosclerosis who experienced incident CVD events, the regression model included respondents with incident CVD events and atherosclerosis, and age, gender, dyslipidemia, circulation problems, diabetes mellitus, hypertension, and smoking were independent variables. A second regression model was computed with respondents with incident CVD events as the dependent variable and atherosclerosis (yes/no), age, gender, dyslipidemia, circulation problems, diabetes, hypertension, and smoking as independent variables to ascertain the value of self-reported atherosclerosis for predicting incident CVD events. Statistical significance was set *a priori *at p < 0.05. The regression model c-statistic was computed to assess goodness of fit for each model.

## Results

### Prevalence and Characteristics of Subclinical Atherosclerosis: Cross-sectional analysis

The prevalence of subclinical atherosclerosis was 2.8% in the baseline survey (488 individuals out of 17,640 respondents). Of the 17,640 respondents, 13,596 did not report a diagnosis of atherosclerosis or prior CVD event and were classified as the comparison cohort, and 3,556 reported having a prior CVD event and were excluded. Individuals reporting an atherosclerosis diagnosis were significantly older, male, less educated, and had lower income than respondents without atherosclerosis diagnosis or CVD event history (p < 0.01) (Table 1). Those reporting subclinical atherosclerosis were also more likely to report dyslipidemia, circulation problems, diabetes mellitus, hypertension, abdominal obesity, a cholesterol test in the past year, or past smoking.

**Table 1:** Comparison of respondents reporting diagnosis of atherosclerosis without history of CVD event in the baseline survey versus respondents with no diagnosis of atherosclerosis and no history of CVD event

**Characteristics**	**Atherosclerosis at baseline** **(n = 488)**	**No atherosclerosis at baseline** **(n = 13,596)**
**Age, years, mean (SD)**	66.9 (12.8)*	51.3 (15.6)

**Men, n (%)**	276 (56.6%)*	4885 (35.9%)

**Race, n (%)**		
**White**	431 (88.3%)	11,779 (86.6%)
**Black**	34 (7.0%)	1025 (7.5%)
**Other**	9 (1.8%)	384 (2.8%)

**Income, n (%)**		
**>$22,500-$39,999**	259 (53.1%)*	5718 (42.1%)
**$40,000-$89,999**	153 (31.3%)	5144 (37.8%)
**≥$90,000**	76 (15.6%)	2734 (20.1%)

**Geographic region, n (%)**		
**Northeast**	116 (23.8%)*	2612 (19.2%)
**North Central**	120 (24.6%)	3372 (24.8%)
**South Atlantic**	108 (22.1%)	2592 (19.1%)
**South Central**	85 (17.4%)	2258 (16.6%)
**Mountain**	19 (3.9%)	901 (6.6%)
**Pacific**	40 (8.2%)	1861 (13.7%)

**Education, n (%)**		
**<8^th ^grade-High school GED**	174 (35.8%)*	3949 (29.4%)
**Some college-college graduate**	253 (52.1%)	7660 (57.0%)
**Graduate degree**	59 (12.1%)	1835 (13.6%)

**Dyslipidemia diagnosis, n (%)**	401 (82.2%)*	6501 (47.8%)

**Circulation problem diagnosis, n (%)**	201 (41.2%)*	1184 (8.7%)

**Diabetes diagnosis, n (%)**	180 (36.9%)*	4243 (31.2%)

**Hypertension diagnosis, n (%)**	375 (76.8%)*	5796 (42.6%)

**BMI category, n (%)**		
**Underweight**	2 (0.4%)	175 (1.3%)
**Normal weight**	71 (14.8%)*	2889 (21.6%)
**Overweight**	157 (32.7%)	3893 (29.1%)
**Obese**	250 (52.1%)	6417 (48.0%)

**Abdominal obesity (waist circumference), n (%)†**	386 (85.4%)*	8948 (70.9%)

**Cholesterol test in past year, n (%)†**	447 (94.3%)*	8943 (71.1%)

**Smoking, n (%)**		
**Current smoker**	83 (17.4%)	2416 (18.0%)
**Past smoker**	235 (49.2%)*	3848 (28.7%)
**Never smoked**	160 (33.5%)	7147 (53.3%)

*Chi-square p < 0.01; †Group n slightly less than other variables

BMI = body mass index; CVD = cardiovascular disease; GED = general equivalency diploma

In the multivariate model, subclinical atherosclerosis was associated with advancing age, male gender, geographic region, dyslipidemia, circulation problems, hypertension, and smoking (Table 2). The model c-statistic was 0.964 (p < 0.05). The odds of atherosclerosis increased by 60% with each decade of increasing age and were twice as high in men. Most US geographic regions had more than twice the odds of atherosclerosis compared with the Pacific region. Respondents with dyslipidemia were 2 times and those with circulation problems were 5 times more likely to have subclinical atherosclerosis than those without these comorbid conditions. Respondents with hypertension or who were smokers (current or past) had a 40%-74% increased odds of having atherosclerosis. Individuals with diabetes mellitus were less likely to have atherosclerosis. Race, household income, education, abdominal obesity, and having a recent cholesterol test were not predictive of atherosclerosis (p > 0.05 for all).

**Table 2:** Logistic regression odds ratios for predictors of subclinical atherosclerosis (no history of prior CVD event) among SHIELD respondents (n = 9,672)

**Characteristics**	**Odds Ratio (95% CI)**	**P value**
**Age (per year)**	1.06 (1.05-1.07)	<0.0001

**Gender, male (female is reference group)**	2.24 (1.80-2.80)	<0.0001

**Race, white (Other is reference group)**	0.65 (0.32- 1.34)	0.24

**Race, black (Other is reference group)**	0.69 (0.31- 1.55)	0.37

**Income, <$22,500 (≥$90,000 is reference group)**	1.03 (0.72- 1.48)	0.87

**Income, $22,500-$39,999**	1.04 (0.73- 1.48)	0.83

**Income, $40,000-$59,999**	0.94 (0.65- 1.35)	0.74

**Income, $60,000-$89,999**	0.91 (0.63- 1.31)	0.61

**Geographic region, New England (Pacific is reference group)**	2.08 (1.17- 3.69)	0.013

**Geographic region, Middle Atlantic**	2.64 (1.71- 4.09)	<0.0001

**Geographic region, East North Central**	2.40 (1.56- 3.69)	<0.0001

**Geographic region, West North Central**	1.59 (0.89- 2.83)	0.12

**Geographic region, South Atlantic**	2.06 (1.35- 3.14)	0.001

**Geographic region, East South Central**	2.53 (1.48- 4.35)	0.001

**Geographic region, West South Central**	1.96 (1.21- 3.17)	0.006

**Geographic region, Mountain**	1.24 (0.67- 2.30)	0.49

**Education, 8^th ^grade (graduate degree is reference group)**	1.08 (0.50- 2.32)	0.85

**Education, some high school**	1.04 (0.58- 1.86)	0.90

**Education, high school graduate**	1.29 (0.88- 1.89)	0.19

**Education, some college**	1.40 (0.98- 2.00)	0.06

**Education, college graduate**	1.05 (0.71- 1.55)	0.80

**Dyslipidemia (cholesterol problems), Yes (No is reference group)**	2.04 (1.56- 2.69)	<0.0001

**Circulation problems, Yes (No is reference group)**	4.95 (3.95- 6.21)	<0.0001

**Diabetes, Yes (No is reference group)**	0.79 (0.64- 0.99)	0.038

**Hypertension, Yes (No is reference group)**	1.74 (1.36- 2.24)	<0.0001

**Abdominal obesity (waist circumference), Yes**	0.80 (0.59- 1.08)	0.14

**Current smoker (Never smoked is reference group)**	1.40 (1.02- 1.91)	0.037

**Past smoker**	1.47 (1.16-1.86)	0.002

**Cholesterol test in past year (Never is reference group)**	1.23 (0.35-4.32)	0.75

Model correctly identified 96.4% of atherosclerosis respondents (c-statistic).

CVD = cardiovascular disease

### Incident CVD Events and Predictors: Longitudinal analysis

A total of 7,483 respondents completed the baseline, 2005, and 2006 surveys and answered the questions on atherosclerosis and CVD events. Among this sample of respondents, 291 individuals had subclinical atherosclerosis at baseline. Of the 291 individuals with atherosclerosis, 75 (25.8%) respondents reported an incident CVD event during the following 2 years. For the respondents without atherosclerosis at baseline (n = 7,192), 878 (12.2%) individuals had an incident CVD event over the 2 years. The relative risk of incident CVD event after adjustment for age, gender, comorbid conditions, and smoking was 2.9 (95% CI: 2.2- 3.85) for those reporting atherosclerosis.

Among individuals who experienced a CVD event during the 2-year follow-up, a significantly greater proportion of respondents with subclinical atherosclerosis were men, and reported a diagnosis of dyslipidemia, circulation problems, or hypertension compared with respondents without subclinical atherosclerosis (p < 0.05) (Table 3). Significantly fewer respondents with subclinical atherosclerosis reported diabetes mellitus compared with respondents without clinical atherosclerosis (p < 0.05). Most respondents (>83%) reported having a cholesterol test in the past 12 months; however, significantly more respondents with atherosclerosis and incident CVD event had a cholesterol test (93%) (p < 0.05). There was no difference between respondents with subclinical atherosclerosis and individuals without atherosclerosis for race, household income, geographic region, obesity, or smoking (Table 3).

**Table 3:** Comparison of respondents with and without subclinical atherosclerosis at baseline but who experience an incident CVD event during 2 years of follow-up

**Characteristics**	**Atherosclerosis at baseline + incident CVD event** **(n = 75)**	**No Atherosclerosis at baseline + incident CVD event** **(n = 878)**
**Age, years, mean (SD)**	68.1 (11.2)*	59.0 (14.5)

**Male, n (%)**	44 (58.7%)*	303 (34.5%)

**Race, n (%)**		
**White**	64 (85.3%)	748 (85.2%)
**Black**	3 (4.0%)	79 (9.0%)
**Other**	2 (2.7%)	14 (1.6%)

**Income ≥$40,000, n (%)**	38 (50.7%)	408 (46.5%)

**Geographic region, n (%)**		
**Northeast**	13 (17.3%)	189 (21.5%)
**South Atlantic**	17 (22.7%)	189 (21.5%)
**Central**	35 (46.7%)	357 (40.7%)
**Mountain**	4 (5.3%)	53 (6.0%)
**Pacific**	6 (8.0%)	90 (10.3%)

**Dyslipidemia diagnosis, n (%)**	61 (81.3%)*	549 (62.5%)

**Circulation problem diagnosis, n (%)**	39 (52.0%)*	136 (15.5%)

**Diabetes diagnosis, n (%)**	22 (29.3%)*	378 (43.1%)

**Hypertension diagnosis, n (%)**	60 (80.0%)*	565 (64.4%)

**BMI category, n (%)**		
**Underweight**	0	9 (1.1%)
**Normal weight**	8 (11.1%)	123 (14.4%)
**Overweight**	30 (41.7%)	237 (27.7%)
**Obese**	34 (47.2%)	486 (56.8%)

**Abdominal obesity (waist circumference), n (%)†**	43 (84.3%)	385 (79.4%)

**Cholesterol test in past year, n (%)†**	67 (93.1%)*	670 (83.1%)

**Smoking, n (%)**		
**Current smoker**	15 (20.0%)	152 (17.5%)
**Past smoker**	34 (45.3%)	304 (35.1%)
**Never smoked**	26 (34.7%)	411 (47.4%)

*p < 0.05; †Group n slightly less than other variables

BMI = body mass index; CVD = cardiovascular disease

**Figure 1:**
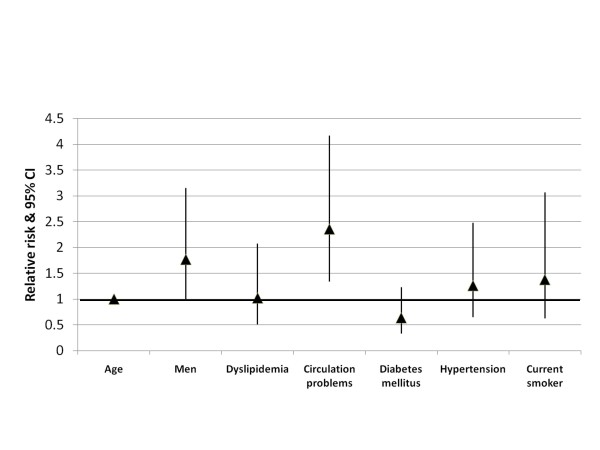
**Relative risk and 95% confidence interval of an incident CVD event among individuals with subclinical atherosclerosis**. Log likelihood = 313.8, R-square = 0.068

Multivariate logistic regression analyses of those with subclinical atherosclerosis demonstrated that men with atherosclerosis had a 77% greater risk of incident CVD event than women (p = 0.05) (Figure 1). The risk of incident CVD events increased 2.4 times among those with circulation problems compared with respondents without circulation problems (p = 0.003) (Figure 1). In a second logistic regression model which assessed the value of self-reported atherosclerosis for predicting incident CVD events, the presence of atherosclerosis increased the risk of incident CVD events by 2.9 times (p < 0.001) while controlling for other covariates. Increasing age (RR = 1.01 per year), type 2 diabetes (RR = 1.37), and hypertension (RR = 1.54) also significantly increased the risk of CVD events in this model (p < 0.01) while gender, dyslipidemia, circulation problems, and smoking were not significant predictors after adjusting for atherosclerosis status.

## Discussion

Several characteristics, easily obtained through self-report, distinguished respondents with subclinical atherosclerosis. Older age, male gender, and smoking were predictive of respondents with subclinical atherosclerosis. Clinically, having dyslipidemia, circulation problems, or hypertension was predictive of individuals with subclinical atherosclerosis. All of these factors can be easily and routinely assessed by primary care physicians to detect those at risk of subclinical atherosclerosis. If management of risk factors and treatment of comorbid conditions are implemented early in the process, atherosclerosis may be delayed and CVD events avoided. Subclinical atherosclerosis was also reported more often in East Coast and Midwest regions than in the Pacific regions, suggesting differential rates of imaging across the US. Several other studies of subclinical atherosclerosis imaging, including coronary artery calcium and carotid artery assessment, have had similar findings. [13,14] However, the role of these techniques in clinical practice has not yet been defined.

The incidence of CVD events was 3 times higher in respondents with subclinical atherosclerosis compared with individuals without atherosclerosis after adjustment for self-reported risk factors. Male gender, dyslipidemia, circulation problems, and hypertension distinguished respondents with subclinical atherosclerosis from individuals without atherosclerosis who later experienced an incident CVD event. Male gender and circulation problems were significant predictors of increased risk of incident CVD events in those reporting subclinical atherosclerosis. Utilizing these easily obtainable characteristics, physicians may identify individuals at risk for CVD event who may be candidates for more aggressive diagnostic and therapeutic interventions.

The prevalence of self-reported atherosclerosis was lower in this study than that found in other studies. [15] The Multi-ethnic Study of Atherosclerosis (MESA) reported a prevalence of 34% based on plaque occurrence and 42% based on calcium score. [15] It is not surprising that the prevalence rates differ since angiographic studies are significantly more sensitive and accurate in quantifying atherosclerosis, especially subclinical atherosclerosis. However, angiographic studies using computed tomography, magnetic resonance imaging, or ultrasound and calcium scores are not done widely in routine clinical practice. The prevalence estimate of atherosclerosis from this study is an under-estimate of the true prevalence.

There are limitations to this study that should be considered. Household panels tend to under-represent the very wealthy and the very poor segments of the population and do not include military or institutionalized individuals. In addition, the determination of atherosclerosis, CVD event, dyslipidemia, hypertension, circulation problems, and diabetes was made based upon self-report rather than clinical or laboratory measures. Nor was the method of assessment available for those reporting subclinical atherosclerosis. Fatal CVD events could not be ascertained in this survey, so the incidence of CVD events may be under-estimated in this study. The proportion of respondents reporting a diagnosis of atherosclerosis was low in this study; many more respondents may have had subclinical atherosclerosis that was not symptomatic or diagnosed.

## Conclusion

Atherosclerosis is a chronic and progressive disease whose optimal prevention requires lifelong attention to diet, exercise, smoking abstinence, and aggressive risk factor identification and treatment. Several characteristics, easily obtained through self-report, may distinguish respondents more likely to have subclinical atherosclerosis. Self-report of subclinical atherosclerosis identifies individuals at very high CVD risk who are candidates for more aggressive risk factor intervention, including cholesterol and blood pressure reduction, management of blood glucose, and weight loss.

## Competing interests

SHIELD is supported by funding from AstraZeneca Pharmaceuticals LP.

JGR is an advisory board member who received an honorarium from AstraZeneca Pharmaceuticals LP. KMF received research funds from AstraZeneca Pharmaceuticals LP to conduct the analysis and prepare the manuscript. MFB and SG are employees and stockholders of AstraZeneca LP.

## Authors' contributions

JGR participated in the design of the study and helped to draft the manuscript. KMF participated in the design of the study, managed the statistical analysis, and drafted the manuscript. MFB assisted with drafting the manuscript. SG conceived of the study, coordinated the data collection, and assisted in drafting the manuscript. All authors read and approved of the final manuscript.

## Pre-publication history

The pre-publication history for this paper can be accessed here:


